# ASO Author Reflections: Postoperative Complications are not Associated with Decreased Health-Related Quality of Life in Patients Following Esophagectomy for Esophageal or Gastroesophageal Junction Cancer

**DOI:** 10.1245/s10434-021-10234-4

**Published:** 2021-06-04

**Authors:** Egle Jezerskyte, Mark I. van Berge Henegouwen, Mirjam A. G. Sprangers, Suzanne S. Gisbertz

**Affiliations:** 1 Department of Surgery, Cancer Center Amsterdam, Amsterdam UMC, University of Amsterdam, Amsterdam, The Netherlands; 2 Department of Medical Psychology, Amsterdam UMC, University of Amsterdam, Amsterdam, The Netherlands

## Past

Esophagectomy has major effects on health-related quality of life (HRQoL), and postoperative complications might contribute to decreased HRQoL. Overall, an impaired short- and long-term HRQoL is reported by patients with postoperative complications compared with patients without postoperative complications following an esophagectomy.^1^–^4^ However, previous studies either did not include a baseline measurement or information on (neo)adjuvant treatment, were performed before the implementation of minimally invasive surgery, or the study was conducted in a single center with a limited number of patients. In the present study,^5^ we dealt with these objections.

## Present

In the current population-based prospective cohort study,^5^ we hypothesized that postoperative complications negatively influence short- and long-term HRQoL. This study targeted 486 patients with esophageal or gastroesophageal junction cancer who were treated with esophagectomy with, among others, minimally invasive surgery and neoadjuvant/perioperative therapy. We found that in general, postoperative complications were not associated with decreased short- and long-term HRQoL in patients up to 24 months post-esophagectomy. We also found a significant decline in short-term HRQoL in patients with or without complications in various HRQoL domains, which restored to baseline level at 12 months of follow-up. No significant difference was found in HRQoL between patients with and without anastomotic leakage. Patients with grade 2–3 anastomotic leakage reported significantly more ‘choking when swallowing’ at 6, 9, and 24 months than patients with grade 1 or no anastomotic leakage.

## Future

Contrary to our expectations, these results suggest that the occurrence of postoperative complications did not influence short- and long-term HRQoL in patients with esophageal or gastroesophageal junction cancer after surgery. We encourage clinicians to inform patients about the significant decline in short-term HRQoL that restores to baseline level at 12 months of follow-up. The temporary decrease in HRQoL was probably related to the nature of the esophagectomy and reconstruction itself. These findings could stimulate future studies to focus on how to minimize the temporary functional complaints caused by esophageal cancer surgery with gastric tube reconstruction.
